# Using knowledge brokers to facilitate the uptake of pediatric measurement tools into clinical practice: a before-after intervention study

**DOI:** 10.1186/1748-5908-5-92

**Published:** 2010-11-23

**Authors:** Dianne J Russell, Lisa M Rivard, Stephen D Walter, Peter L Rosenbaum, Lori Roxborough, Dianne Cameron, Johanna Darrah, Doreen J Bartlett, Steven E Hanna, Lisa M Avery

**Affiliations:** 1 CanChild Centre for Childhood Disability Research, McMaster University, Hamilton, Ontario, Canada; 2 School of Rehabilitation Science, McMaster University, Hamilton, Ontario, Canada; 3 Department of Clinical Epidemiology and Biostatistics, McMaster University, Hamilton, Ontario, Canada; 4 Department of Pediatrics, McMaster University, Hamilton, Ontario, Canada; 5 Department of Occupational Science and Occupational Therapy, University of British Columbia, Vancouver, British Columbia, Canada; 6 Department of Therapy Services, BC Centre for Ability, Vancouver, British Columbia, Canada; 7 Department of Physical Therapy, University of Alberta, Edmonton, Alberta, Canada; 8 School of Physical Therapy, The University of Western Ontario, London, Ontario, Canada

## Abstract

**Background:**

The use of measurement tools is an essential part of good evidence-based practice; however, physiotherapists (PTs) are not always confident when selecting, administering, and interpreting these tools. The purpose of this study was to evaluate the impact of a multifaceted knowledge translation intervention, using PTs as knowledge brokers (KBs) to facilitate the use in clinical practice of four evidence-based measurement tools designed to evaluate and understand motor function in children with cerebral palsy (CP). The KB model evaluated in this study was designed to overcome many of the barriers to research transfer identified in the literature.

**Methods:**

A mixed methods before-after study design was used to evaluate the impact of a six-month KB intervention by 25 KBs on 122 practicing PTs' self-reported knowledge and use of the measurement tools in 28 children's rehabilitation organizations in two regions of Canada. The model was that of PT KBs situated in clinical sites supported by a network of KBs and the research team through a broker to the KBs. Modest financial remuneration to the organizations for the KB time (two hours/week for six months), ongoing resource materials, and personal and intranet support was provided to the KBs. Survey data were collected by questionnaire prior to, immediately following the intervention (six months), and at 12 and 18 months. A mixed effects multinomial logistic regression was used to examine the impact of the intervention over time and by region. The impact of organizational factors was also explored.

**Results:**

PTs' self-reported knowledge of all four measurement tools increased significantly over the six-month intervention, and reported use of three of the four measurement tools also increased. Changes were sustained 12 months later. Organizational culture for research and supervisor expectations were significantly associated with uptake of only one of the four measurement tools.

**Conclusions:**

KBs positively influenced PTs' self-reported knowledge and self-reported use of the targeted measurement tools. Further research is warranted to investigate whether this is a feasible, cost-effective model that could be used more broadly in a rehabilitation setting to facilitate the uptake of other measurement tools or evidence-based intervention approaches.

## Background

'Best practice' is defined as the integration of research evidence, client preferences, and clinical experience [1]. In pediatric physical therapy, clinical practice includes examination and evaluation of the client, diagnosis, prognosis, intervention, and evaluation of outcomes [2]. All of these components of practice require documentation and measurement. Standardized measures assist physiotherapists (PTs) to assess children's abilities, limitations and potential objectively. Clinical use of reliable and valid outcome measures also facilitates collaborative clinical and administrative decision-making, and evaluation of change of children's abilities. For research purposes, aggregation of data from standardized measures allows for evaluation of intervention outcomes.

**Figure 1:**
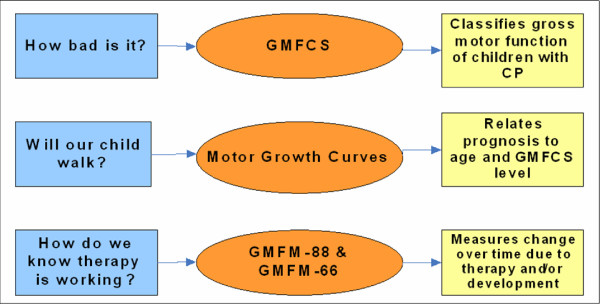
**Parents questions and how the motor measures can help**. GMFCS = Gross Motor Function Classification System, GMFM = Gross Motor Function Measure, CP = Cerebral Palsy.

Investigators at *CanChild *Centre for Childhood Disability Research at McMaster University, Ontario, Canada have developed and validated a set of measurement tools to assist in the measurement and understanding of gross motor function of children with cerebral palsy (CP). These tools include the Gross Motor Function Classification System (GMFCS) [3,4], the Gross Motor Function Measure (GMFM-88 [5] and GMFM-66 [6-8]), and the Motor Growth Curves (MGCs) [9]. When used together, this collection of tools provides an integrated, evidence-based approach to clinical practice and can help service providers set and evaluate intervention goals and answer parents' questions about prognosis (Figure 1). These measurement tools are recognized internationally in the research/academic community as the gold standard measures of motor function for children with CP. Our group has extensive experience training clinicians to use these tools [10,11] and continues to research the development and application of the tools in research and clinical practice [12-19].

Although PTs recognize the importance of using standardized measures as part of evidence-based practice, they face many challenges in selecting, using and interpreting the information from measures [20]. The challenges to moving outcome measures into clinical practice in children's rehabilitation settings [20,21] are similar to those reported in a systematic review of barriers to moving evidence to practice more broadly [22]. Specifically, Cochrane *et al*. [22] identified seven categories of barriers: supports/resources (*e.g*., time, funding, resources), cognitive/behavioural (*e.g*., knowledge, awareness, skills), healthcare professional (*e.g*., characteristics, age/maturity of practice, peer influence), system/process (*e.g*., workload, team structure, referral process), attitudinal/rational-emotive (*e.g*., perceived competence, perceived outcome expectancy, authority), clinical practice guidelines/evidence (*e.g*., utility, access, local applicability), and patient factors (*e.g*., patient characteristics, adherence).

A survey of pediatric PTs and occupational therapists (OTs) [23] revealed wide variation in the practices of therapists treating young children with CP in relation to best practice guidelines. One solution suggested by the authors was the promotion of knowledge translation (KT) strategies to encourage evidence-based practice among practicing clinicians. The Canadian Institutes of Health Research defines KT as a 'dynamic and iterative process that includes synthesis, dissemination, exchange and ethically sound application of knowledge to improve health, provide more effective health services and products and strengthen the healthcare system' [24].

Researchers are now beginning to investigate KT strategies within clinical contexts.

In a study examining the use of outcome measures by pediatric PTs in the Netherlands, passive KT strategies such as peer-reviewed journal articles and web-based summaries were found to be effective in increasing awareness of outcome measures, but did not increase their use [25]. Although interactive workshops were somewhat successful in increasing use of the measures, a significant gap still remained between knowledge and use. It was proposed that ongoing support and opportunities to share experiences with peers may be necessary for the clinical use of evidence-based measures to be maintained long term.

In a clinical trial, mental health practitioners randomized to a 'community of practice' group demonstrated more frequent clinical use of an accepted standardized measure compared to those who had access only to their organizations' regular supports [26]. Communities of practice may be an effective KT strategy to support use of research evidence in clinical practice. In addition to the interactive aspect, this approach also involves individuals from within the clinical practice setting. Because barriers to using evidence in practice will vary by practice setting, having someone from within the clinical practice setting available to help identify both barriers and supports would be important to influence effective KT strategies.

A recent systematic review of strategies used by rehabilitation professionals to move evidence into practice suggested that active, multi-component interventions improve evidence-based knowledge and behaviours by PTs [27]. One strategy for knowledge transfer that is gaining interest is a KT program built around the roles and activities of a knowledge broker (KB). A KB has been defined as someone who is capable of 'bringing researchers and decision makers together, facilitating their interaction so that they are able to better understand each others' goals and professional culture, influence each others' work, forge new partnerships, and use research-based evidence. Brokering is ultimately about supporting evidence-based decision making in the organization, management and delivery of health services' [28]. Pediatric PTs in children's rehabilitation settings share common values, interests, and uncertainties about their work, and within these communities of practice the role of the KB may be particularly useful.

Despite the increasing interest in knowledge brokering, little research evidence exists regarding the use of a KB. A common feature among different types of brokering models is the concept of interactive engagement; however, the specific brokering activities of the KB are difficult to define or standardize because the role should be flexible and responsive to the needs of the stakeholders [29,30]. Most brokering studies to date have been in policy decision-making environments [31-33]. Although there is evidence that KBs help decision makers gain knowledge and skills in the evidence-based process [32], Dobbins *et al*. [29] found that the use of a KB in addition to tailored messages linking relevant research evidence to specific decision makers was not as effective as tailored messages alone in influencing policy decision making for public health organizations with a high research culture. These findings are useful; however, the environments in which policy makers and front-line clinicians practice are very different, as are the issues that they must address. Thus, investigation of a KB model in a clinical environment was warranted.

The primary purpose of this study was to evaluate the short-term (six-month) and long-term (12-month) impact of a multi-faceted KT intervention using KBs to facilitate the use of four evidence-based measurement tools by PTs in children's rehabilitation facilities in Ontario (the 'East'), and Alberta and British Columbia (the 'West'). A secondary purpose of the study was to explore factors such as organizational support that might modify or mediate the intervention.

We hypothesized that in both regions (East and West), PTs would increase their knowledge and use of the measurement tools, but that there would be regional differences because of baseline differences in familiarity with the tools between the regions. Therapists in the East have a longstanding partnership with *CanChild *and their involvement in previous research related to the development and validation of the measurement tools may provide them with more familiarity with the tools. The natural variation between the regions enhances the generalizability of the intervention approach across settings, level of baseline knowledge, and use. We also hypothesized that organizational culture would influence the uptake of evidence based on previous work [31,34].

**Figure 2:**
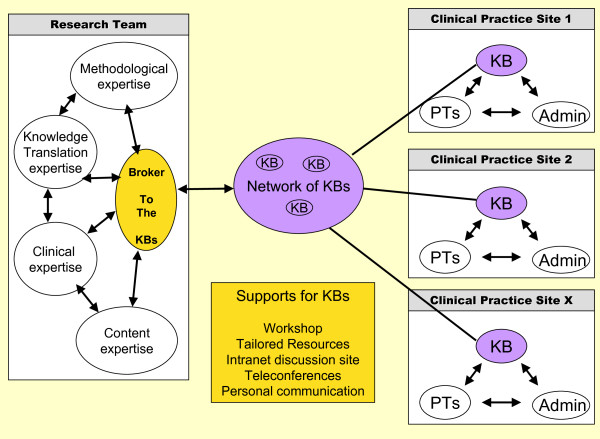
**A broker to the knowledge brokers (KB) model**. This figure illustrates our model of a KB situated in the clinical practice site who is working to facilitate the uptake of the measurement tools in their practice site. The KBs are assisted in their role by the research team including a Broker to the KBs, by a network of all the KBs and additional personal and resource supports. KB = Knowledge Broker, PTs = Physiotherapists, Admin = Administration.

We developed a KT model of a KB embedded within the clinical context and supported by the network of knowledge brokers and the research team (including a broker to the knowledge brokers). We refer to this as a 'broker to the knowledge brokers' model (Figure 2). This model focused on the 'action' or implementation phase of the knowledge to action (KTA) framework [35], with an emphasis on knowledge uptake rather than on providing a synthesis of the evidence or on teaching clinicians to be experts in critical appraisal.

## Methods

### Design

A mixed methods, before-after study design was used to evaluate the impact of a six-month multi-faceted KB intervention on PTs' self-reported knowledge and use of the motor measurement tools measured using an on-line survey questionnaire. Follow-up questionnaire data were collected from PTs immediately following the intervention (six months after baseline) and at 12 and 18 months to examine the long-term impact. The KB process was also evaluated using systematic documentation of activities (log books) employed by KBs throughout the study, and semi-structured telephone interviews conducted with multiple stakeholders (KBs, PTs, and organization administrators) immediately post-intervention and one year later to evaluate the perceived utility of the KB intervention [36]. The focus of this paper is on the questionnaire results on familiarity and use of the measurement tools.

### Sample size justification

Sample size calculations made prior to the study were based on the power to detect change in the primary outcome measure between any two of the intervention points. Estimates were conservatively large, based on a test-retest reliability of 0.50 (ICC), a strong cluster effect due to centres (ICC = 0.20) and an average of three therapists per centre, with a 5% Type I error rate. Varying these assumptions, it was estimated that we would have at least 80% power to detect a standardized change of 0.19 standard deviations or more. The target number of PTs required (not including knowledge brokers) was 90.

### Setting and participants

Children's rehabilitation organizations provide therapy services (physiotherapy, occupational therapy, and speech and language pathology) for children from birth to 19 years old with physical, developmental, and/or communication difficulties. Participating centres represented both rural and urban settings and large and small centres. Organizations involved in this study provided services to children in a variety of settings including on site, in preschools and schools, at home, and in the community. The administrators and PT managers of 35 children's rehabilitation organizations were invited to participate. The inclusion criteria specified the need to have at least three PTs working at the site, in order to have one therapist take on the role of the KB and have at least two therapists to participate in the brokering process. Three sites were very eager to participate but did not have three PTs. These sites were subsequently included as 'regional' sites, using a KB from another participating centre who agreed to broker to them in addition to their own site. Twenty-eight children's rehabilitation organizations participated in the study, with 16 centres in the East (Ontario), and 12 in the West (two in Alberta, and 10 in British Columbia).

Twenty-five pediatric PT KBs were recruited from among the staff at the participating sites. KBs applied, were chosen, or volunteered for the role, based on a number of factors including their interest in and enthusiasm for the role, as well as their ability to adjust their schedule to accommodate the requirements of the study (two hours/week for six months). Twenty-four of the 25 KBs remained in the study for the 18-month duration. One KB in the West changed employment shortly after baseline, and one of the KBs in geographic proximity agreed to become a regional broker for this site. KBs were generally experienced clinicians with 19 (80%) having 10 or more years working in childhood disability, and three (13%) having less than five years in pediatric practice. Sixteen KBs (67%) worked in urban settings (population >100,000) and spent, on average, 24% of their working time in direct patient care.

The target of the KB intervention was practicing PTs who currently had (or anticipated having) at least three children with CP on their caseload during the six months of the brokering intervention. A total of 122 therapists consented to participate and completed the baseline questionnaire. Ninety (74%) PTs had five or more years experience working in childhood disability, and 88 (72%) worked in urban centres with only one therapist working in a remote area (population <3,000). PTs spent on average 53% of their time in direct care. Table 1 shows the demographic characteristics of the PTs and Table 2 details the number of therapists in each region who responded to the online survey questionnaire at each of the four time points. Overall, 95 PTs (78%) remained in the study for the 12-month follow-up. The number of PTs brokered to at each site varied, with one to two therapists at each of the three regional sites and three to nine therapists at the remaining sites.

**Table 1:** Demographic characteristics of physical therapists (PTs) (n = 122)

	n (%)
**Province of Practice**	
East (Ontario)	71 (58.2)
West (Alberta and British Columbia)	51 (41.8)
	
**Length of employment at current practice site**	
Less than 1 year	15 (12.3)
1 year to <5 years	36 (29.5)
5 years to < 10 years	21 (17.2)
10 years or longer	50 (41.0)
	
**Number of years working in childhood disability**	
Less than 1 year	8 (6.6)
1 year to <5years	24 (19.7)
5 years to <10 years	29 (23.8)
10 years or longer	61 (50.0)
	
**Number of PTs contracted to work in other settings (with children with CP)**	
Community-based	18 (14.8)
Centre/facility-based	4 (3.3)
	
Number of PTs serving children in various age ranges (all settings)*	
Birth to <3 years	87 (71.3)
3 years to <6 years	99 (81.1)
6 years to <12 years	90 (73.4)
12 years or older	79 (64.8)
	
**Percentage of time spent in direct care (mean, (SD))**	53.4 (25.1)
	
**Geographical region predominantly served**	
Urban (population >100,000)	88 (72.1)
Rural (population between 3,000 and 99,999)	33 (27.0)
Remote (population less than 3,000)	1 (0.8)

Note: * PTs could be working with children in more than 1 age group;

PT = physical therapist, CP = cerebral palsy, SD = standard deviation, n = number, % = percent of total

**Table 2:** Number of completed therapist surveys by region for each time point†

	Baseline	6 months	12 months	18 months
East	71	67	67	59

West	51	47	38	36

Total	122	114	105	95

^† ^Sample size reduced by therapists on leave, missing data

### Intervention

The intervention involved pediatric PT KBs situated in clinical sites who were supported by the network of KBs and the research team (Figure 2). Supports for the KBs included access to the study team and research coordinator, an experienced pediatric PT who had used the measurement tools clinically. The research coordinator functioned as a 'broker to the knowledge brokers' providing timely responses to questions and encouraging linkages both among KBs and between the KBs and the researchers. Graham *et al*.'s KTA framework [35] was used to plan the intervention, including adapting knowledge to the local context; assessing barriers and supports; selecting, tailoring, and implementing interventions; and monitoring use and evaluating outcomes. Details of the study activities are described in Table 3. Supportive activities for the KBs included an initial face-to-face one-day interactive workshop with KBs and the study team (many of whom were content experts regarding the measurement tools). It is important to note that in the workshop KBs were not trained on the measurement tools themselves but used small group sessions to discuss the roles and responsibilities of the KB and possible KT strategies. In addition, they were provided with information about the central supports available to the KBs through the study team.

**Table 3:** Design of knowledge brokering intervention (based on the KTA framework 35)

Phase 1: Adapting knowledge to local context - Knowledge tools and products	Content-specific materials synthesized, tailored for easy KB access
	Pre-workshop package sent to KBs containing a GMFM-88/66 manual, instructional CD-ROM, GMFCS training DVD, key published articles, and user-friendly summaries and case scenarios
	Additional materials posted on a private 'KB Discuss' intranet site
	Intranet site designed so KBs could post and respond to questions - community of practice encouraged
	Intranet site moderated by research team
	Power-Point presentations about the measures made available for download (KBs encouraged to modify and tailor)
**Phase 2:** **Assessing barriers and supports**	In preparation for the KB interactive workshop, KBs completed a 'Supports and Barriers Questionnaire' to identify possible supports and barriers to implementation of the motor measures within their own clinical context.
	They were asked to consider factors within their organizational structure and resources, the target therapists, the measures themselves, and the children and families

**Phase 3:** **Selecting, tailoring, and implementing interventions**	KBs empowered to select, tailor, and implement interventions as they felt appropriate
	KBs tracked activities using a weekly log book
	Regular KB teleconferences and use of online 'KBdiscuss' site facilitated sharing of strategies

**Phase 4:** **Monitoring use and evaluating outcomes**	KBs and PTs completed online survey of knowledge and use, pre-brokering, 6, 12, and 18 months
	KBs, PTs, and centre administrators completed a semi-structured telephone interview about the utility of the KB process at 6 and 18 months

In preparation for the workshop, KBs completed a questionnaire about the perceived supports and barriers to moving the measurement tools into practice at their organization. The questionnaire was designed for this study and based on factors identified by Fleuren *et al*. [37] as important determinants of innovation in healthcare organizations. KBs reflected on perceived supports and barriers related to their organizational structure, their organizational resources, their target therapists, the children with CP and their families, and the measurement tools themselves. KBs were provided with tailored resources related to the measurement tools (including user-friendly evidence-based summaries and case scenarios, CD-ROM training materials, *etc*.) for use in the KBs' own site. Rather than overwhelming the KBs with an abundance of information, a private intranet site was set up where additional materials (including prepared slide presentations) were posted for their use and modification as needed. The types of supports and resources accessed by KBs during the study were left to the discretion of the KB based upon the needs and strengths of the KB, their therapists, and their organization. A detailed description of the strategies used and the resources accessed by KBs is reported elsewhere [36], but consisted of activities such as self learning, needs assessments, presentations, group discussions, accessing and modifying resources, one-on-one interactions with various stakeholders, networking with other KBs, accessing computer support, and collaborative measurement and scoring of clients.

Ongoing collaboration amongst KBs was encouraged through an intranet discussion site that was monitored and moderated by members of the study team. Three KB teleconferences were held during the six-month intervention, providing opportunities for KBs to network with each other, and interface with the research team. Release time for the KBs in the form of financial support was provided to organizations for two hours per week during the six-month intervention. KBs were able to use the two hours per week flexibly, depending upon their schedules (*e.g*., not necessarily two hours every week, but to average out to that amount over the six months).

At the end of the six-month brokering period, KBs participated in a face-to-face workshop to discuss preliminary results, provide feedback on current brokering activities, and identify next steps in the research process.

Ethics approval was obtained from research ethics boards at McMaster University, University of Alberta, University of Calgary, and University of British Columbia. Informed consent was obtained from all participating KBs, PTs, and administrators.

### Measures

#### Evaluation of uptake of the measurement tools

The primary outcome was change in PTs' self-reported knowledge and use of the measurement tools assessed using a standardized questionnaire developed for the study. The questionnaire provided ratings of familiarity with and use of the four measurement tools (GMFCS, GMFM-88, GMFM-66, Motor Growth Curves) assessed on a 10-point Likert scale (from 'not at all' to 'to a great extent'). Based on previous work evaluating measures and knowledge uptake [20,31], nine questions concerning organizational support were included in the questionnaire. The questionnaire was pilot-tested with 27 therapists and modified based on their feedback prior to its use in this study. Test-retest reliability of a Dutch translation of the questionnaire found item ICCs ranging from 0.75 to 0.98 for items related to familiarity and use of the measurement tools and from 0.29 to 0.91 for organizational characteristics (Ketelaar, personal communication).

#### Evaluation of KB Process

During the six-month intervention, KBs submitted a weekly log of their activities to the research coordinator. During the 12-month follow-up, KB logs were submitted monthly to document the extent to which brokering activities continued following withdrawal of financial support for the role. The KBs documented the number and type of contacts (*e.g*., contact with PTs involved in the study or others external to the study), who initiated the contacts (*e.g*., the KB or someone from the centre), the type of activity (educational session, case discussion), and the format of the activities (*e.g*., face-to-face meeting, individual or group session). In addition they documented the supports they accessed (*e.g*., the intranet site, other KBs, technical support) and indicated any resources they developed (*e.g*., flyers, surveys).

### Analysis

#### Evaluation of uptake of the measurement tools

To examine uptake of the measurement tools by PTs, eight outcomes were investigated and included 'familiarity' and 'use' for each of the four tools, scored on a 10-point Likert scale. Because the data were not normally distributed (some outcomes were bimodal, some severely skewed) and to facilitate clinical interpretation, the original 10-point scale was collapsed into three categories (1 = 'none'; 2 to 7 = 'some'; 8 to 10 = 'high'; with 'some' as the reference category). It was felt that moving from being a 'non-user' of a measure to being a user was a more significant change than a change of one or two points in original scale (*i.e*., a change in the original scale might be more difficult to interpret than a change between 'levels' or categories of the outcome). Where there were too few 'none' responses, the 'none' and 'some' categories were combined.

Mixed-effects multinomial logistic regression was used with the MIXNO program [38] to examine the impact of the intervention by making comparisons over time and investigating the effect of region (East or West) on the outcomes. Multinomial, as opposed to ordinal logistic regression was used because the assumption of proportional odds was violated. Therapists and site were modelled as random effects and time and region were modelled as fixed effects. Because each site had only a single KB, either site or KB could be included in the model, and we chose to include the site. The effect of organizational characteristics (overall culture and supervisor expectation) was also investigated to determine if they improved the model fit for the outcomes of interest.

#### Organizational culture

PTs answered nine questions about the organizational characteristics and culture towards research and evidence-based practice within their organizations. Each question was scored on a 10-point scale with response options ranging from 'not at all' to 'to a great extent.' Factor analysis of the items was done to determine whether it would be appropriate to combine items into a separate overall 'organizational culture' score.

#### Sensitivity analysis

To determine the potential impact of data collected from PTs who were away or on leave for more than one month during the study period, a sensitivity analysis was completed omitting those PTs (n = 27) from the analysis. Overall, there were no important differences in the results with the data from these PTs removed; therefore all data were included in the final analyses.

## Results

### Familiarity and use of the motor measurement tools

**Figure 3:**
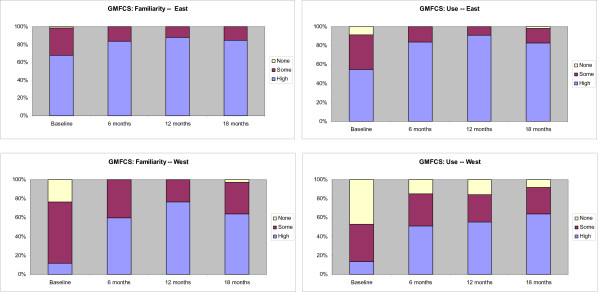
**Changes in the familiarity and use of the Gross Motor Function Classification System (GMFCS) at baseline and at 6-, 12-, and 18-month follow-up**. Stacked bar graphs reflect the percentage of participants reporting none, some, or high familiarity and use of the measure, where none = 1, some = 2 to 7, and high = 8 to 10 on a 10-point Likert scale. Odds ratios are reported in Table 4.

**Figure 4:**
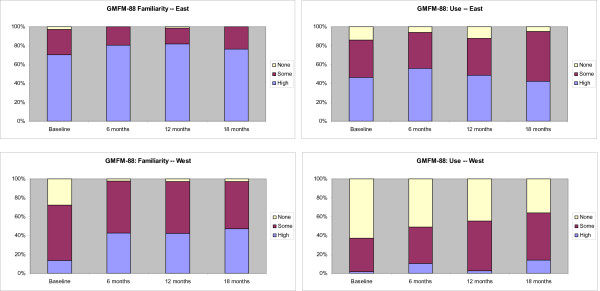
**Changes in the familiarity and use of the Gross Motor Function Measure 88 (GMFM-88) at baseline and at 6-, 12-, and 18-month follow-up**. Stacked bar graphs reflect the percentage of participants reporting none, some, or high familiarity and use of the measure, where none = 1, some = 2 to 7, and high = 8 to 10 on a 10-point Likert scale. Odds ratios are reported in Table 5.

**Figure 5:**
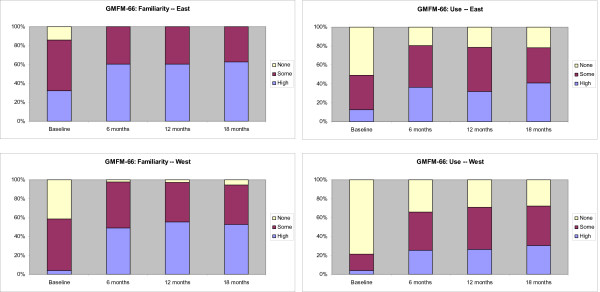
**Changes in the familiarity and use of the Gross Motor Function Measure 66 (GMFM-66) at baseline and at 6-, 12-, and 18-month follow-up**. Stacked bar graphs reflect the percentage of participants reporting none, some, or high familiarity and use of the measure, where none = 1, some = 2 to 7, and high = 8 to 10 on a 10-point Likert scale. Odds ratios are reported in Table 6.

**Figure 6:**
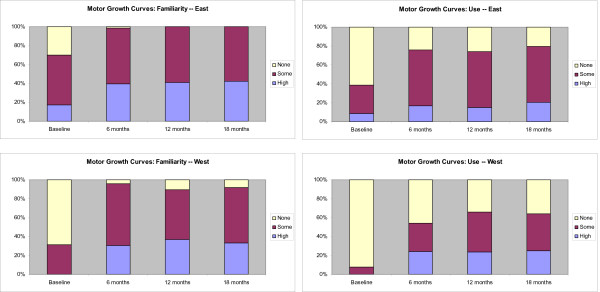
**Changes in the familiarity and use of the Motor Growth Curves (MGCs) at baseline and at 6-, 12-, and 18-month follow-up**. Stacked bar graphs reflect percentage of participants reporting none, some, or high familiarity and use of the measure, where none = 1, some = 2 to 7, and high = 8 to 10 on a 10-point Likert scale. Odds ratios are reported in Table 7.

A description of the four measurement tools and their measurement characteristics is outlined in an Additional file 1, Table S1. The measurement tools have been developed, validated, and published over the past 20 years and vary in their complexity to learn and use. Stacked bar graphs displaying the results are shown in Figures 3, 4, 5, and 6. The conditional odds ratios for each of the measurement tools (GMFCS, GMFM-88, GMFM-66, MGCs), by region over time for the outcomes 'familiarity' and 'use,' are presented in Tables 4, 5, 6, and 7. When a time by region interaction was identified, separate results for East and West are presented; otherwise the data are combined across regions. A few results (*e.g*., in Tables 4 and 6) show high odds ratios with very wide confidence intervals; the instability of these results is due to the small number of therapists in the West who reported high familiarity at baseline.

**Table 4:** Gross Motor Function Classification System (GMFCS) familiarity and use over time

GMFCS Familiarity				GMFCS Use			
EAST^1^				EAST + WEST			
High versus [Some + None]^2^				High versus Some^3^			
	
Time Interval	OR	95% CI	p-value	Time Interval	OR	95% CI	p-value
	
Baseline to 6 mos	7.2	2.0 to 25.9	<0.01	Baseline to 6 mos	18.2	5.5 to 60.1	<0.01
	
6 to 12 mos	1.5	0.2 to 9.5	0.67	6 to 12 mos	1.8	0.3 to 10.6	0.49
	
12 to 18 mos	0.8	0.1 to 6.2	0.84	12 to 18 mos	0.8	0.1 to 4.8	0.80
	
				East versus West	17.7	4.1 to 76.3	<0.01
	

WEST^1^				EAST + WEST			
High versus [Some + None]^2^				Some versus None^3^			
	
Time Interval	OR	95% CI	p-value	Time Interval	OR	95% CI	p-value
	
Baseline to 6 mos	378.1	12.2 to 11676.1	<0.01	Baseline to 6 mos	11.8	2.4 to 57.7	<0.01
	
6 to 12 mos	5.8	0.0 to 1123.6	0.51	6 to 12 mos	0.5	0.0 to 8.8	0.67
	
12 to 18 mos	0.1	0.0 to 31.6	0.48	12 to 18 mos	1.6	0.0 to 50.0	0.80
	
				East versus West	15.0	1.7 to 134.3	0.01
	
High versus [Some + None]^2^				High versus Some^3^			
	
Organizational Characteristics	OR	95% CI	p-value	Organizational Characteristics	OR	95% CI	p-value
	
Research culture	3.6	1.5 to 8.7	0.01	Research culture	1.6	0.8 to 3.2	0.16
	
Supervisor expectation	1.5	0.7 to 2.9	0.29	Supervisor expectation	2.0	1.0 to 3.9	0.04
	
				Some versus None^3^			
	
				Organizational Characteristics	OR	95% CI	p-value
	
				Research culture	3.0	1.1 to 7.9	0.03
	
				Supervisor expectation	2.6	1.1 to 6.0	0.03

^1^Time × Region Interaction;

^2 ^Response Categories = High/Some + None

^3^Response Categories = High/Some/None

OR = odds ratio

CI = confidence interval

**Table 5:** Gross Motor Function Measure (GMFM-88) familiarity and use over time

GMFM-88 Familiarity				GMFM-88 Use			
EAST + WEST				EAST + WEST			
High versus [Some + None]^1^				High versus Some^2^			
	
Time Interval	OR	95% CI	p-value	Time Interval	OR	95% CI	p-value
	
Baseline to 6 mos	6.1	2.3 to 15.8	<0.01	Baseline to 6 mos	2.7	0.9 to 7.8	0.07
	
6 to 12 mos	1.0	0.3 to 3.4	0.95	6 to 12 mos	1.2	0.4 to 3.3	0.73
	
12 to 18 mos	1.0	0.3 to 3.2	0.93	12 to 18 mos	1.2	0.4 to 3.8	0.77
	
East versus West	35.3	8.7 to 143.1	<0.01	East versus West	84.7	11.1 to 644.8	<0.01
	

				EAST + WEST			
				Some versus None^2^			
				
				Time Interval	OR	95% CI	p-value
				
				Baseline to 6 mos	2.2	1.0 to 5.1	0.07
				
				6 to 12 mos	1.0	0.3 to 3.7	0.81
				
				12 to 18 mos	2.2	0.6 to 8.2	0.75
				
				East versus West	13.1	4.5 to 38.3	<0.01

^1^Response Categories = High/Some + None

^2^Response Categories = High/Some/None

OR = odds ratio

CI = confidence interval

**Table 6:** Gross Motor Function Measure (GMFM-66) familiarity and use over time

GMFM-66 Familiarity				GMFM-66 Use			
EAST^1^				EAST + WEST			
High versus [Some + None]^2^				High versus Some^3^			
	
Time Interval	OR	95% CI	p-value	Time Interval	OR	95% CI	p-value
	
Baseline to 6 mos	7.4	2.8 to 19.8	<0.01	Baseline to 6 mos	6.2	2.2 to 17.6	<0.01
	
6 to 12 mos	1.1	0.3 to 4.1	0.89	6 to 12 mos	0.9	0.2 to 4.2	0.87
	
12 to 18 mos	1.0	0.3 to 4.0	0.95	12 to 18 mos	1.7	0.3 to 8.8	0.53
	
				East versus West	1.8	0.6 to 5.3	0.26
				

WEST^1^				EAST + WEST			
High versus [Some + None]^2^				Some versus None^3^			
	
Time Interval	OR	95% CI	p-value	Time Interval	OR	95% CI	p-value
	
Baseline to 6 mos	238.7	6.4 to 8923.9	<0.01	Baseline to 6 mos	7.9	3.7 to 17.0	<0.01
	
6 to 12 mos	1.5	0.0 to 230.5	0.87	6 to 12 mos	1.2	0.4 to 3.8	0.81
	
12 to 18 mos	0.8	0.0 to 114.4	0.94	12 to 18 mos	0.8	0.2 to 2.9	0.75
	
				East versus West	3.3	1.5 to 7.4	<0.01

^1^Time × Region Interaction.

^2 ^Response Categories = High/Some + None

^3^Response Categories = High/Some/None

OR = odds ratio

CI = confidence interval

**Table 7:** Motor Growth Curves familiarity and use over time

Motor Growth Curves Familiarity				Motor Growth Curves Use			
EAST + WEST				EAST + WEST			
High versus Some^1^				High versus Some^1^			
	
Time Interval	OR	95% CI	p-value	Time Interval	OR	95% CI	p-value
	
Baseline to 6 mos	13.9	4.0 to 48.7	<0.01	Baseline to 6 mos	3.3	1.1 to 9.8	0.03
	
6 to 12 mos	1.4	0.3 to 7.1	0.71	6 to 12 mos	0.9	0.2 to 4.3	0.87
	
12 to 18 mos	0.9	0.2 to 4.1	0.89	12 to 18 mos	1.3	0.2 to 6.8	0.79
	
East versus West	2.7	0.7 to 10.8	0.16	East versus West	0.4	0.1 to 1.1	0.07
	

EAST + WEST				EAST + WEST			
Some versus None^1^				Some versus None^1^			
	
Time Interval	OR	95% CI	p-value	Time Interval	OR	95% CI	p-value
	
Baseline to 6 mos	188.6	15.7 to 2269.9	<0.01	Baseline to 6 mos	39.2	10.9 to 140.2	<0.01
	
6 to 12 mos	0.5	0.0 to 13.1	0.70	6 to 12 mos	1.3	0.2 to 8.3	0.75
	
12 to 18 mos	1.3	0.1 to 29.2	0.87	12 to 18 mos	1.2	0.2 to 7.5	0.84
	
East versus West	13.3	3.6 to 48.8	<0.01	East versus West	9.9	2.5 to 39.2	<0.01
	

^1^Response Categories = High/Some/None

OR = odds ratio

CI = confidence interval

### Familiarity and Use of the GMFCS

The GMFCS is a five-level severity classification system and is the easiest of the measurement tools to learn and to use. It can be used by rehabilitation service providers other than PTs and may therefore be of interest to other clinicians and administrators in children's rehabilitation organizations.

The GMFCS was the most familiar of the four measurement tools to therapists in both regions. Therapist reported familiarity and use of the GMFCS over time is displayed in Figure 3. At baseline, 70 (99%) PTs in the East reported having at least some familiarity with the GMFCS and 65 (92%) reported having used it. Following the six-month intervention, all therapists in the East were familiar with the GMFCS and all reported using it at least once. High users increased from 55 to 84%.

In the West, there was a wider gap between familiarity and use of the GMFCS than in the East, with 39 (77%) PTs reporting at least some familiarity with the GMFCS at baseline, and 27 (53%) PTs indicating that they had used it at least once. Immediately post-intervention, all therapists in the West were familiar with the GMFCS and overall use in the West increased from 53% to 85%, with high users increasing from 14% to 51%.

The conditional odds ratios for familiarity and use of the GMFCS are presented in Table 4. There was a time by region interaction for familiarity and therefore results are presented separately for the East and the West. Because there were so few therapists who were not at all familiar with the GMFCS, results for 'no familiarity' were combined with 'some familiarity' and compared to 'high' familiarity. There was a significant increase in therapists' familiarity with the GMFCS at six months compared to baseline (odds ratios of having 'high familiarity' versus 'some familiarity' was 7.2 (95% CI: 2.0 to 25.9) in the East and 378.1 (95% CI 12.2 to 11676.1) in the West. The high odds ratio with very wide confidence intervals in the West is due to the small number of therapists in the West who reported high familiarity at baseline. Results also show that the odds ratios did not change significantly in the 6- or 12-month follow-up, indicating that the change from baseline was maintained.

Looking at GMFCS use there was no interaction so results for East and West are combined. The odds ratio of moving from 'no use' to 'some use' was 11.8 (95% CI 2.4 to 57.7) and from 'some use' to 'high use' of the GMFCS following the intervention was 18.2 (95% CI 5.5 to 60.1). Changes in GMFCS use were maintained at 6 and 12 months. Therapists in the East were 17 times more likely to report high use relative to those in the West and 15 times more likely to report using it 'some' than not using it at all compared to therapists in the West.

### Familiarity and use of the GMFM-88

The GMFM-88 was first published in 1989 and is used to evaluate change in the gross motor abilities of children with CP. Although the GMFM-88 provides a detailed assessment of gross motor skills, it takes time to learn to administer and score its 88 items (*e.g*., typically several hours of reading and practicing). In addition, administering the test with children usually takes 45 to 60 minutes.

At baseline, therapists in the East were very familiar with the GMFM-88, with 69 (97%) PTs reporting they were at least somewhat familiar with the GMFM-88, and 50 (70%) indicating that they were highly familiar (Figure 4). Overall 61 (86%) PTs reported using the GMFM-88 at baseline with 33 (47%) of those indicating high use. Following the six-month intervention, all therapists in the East were familiar with the GMFM-88 and there was no significant increase in reported use from baseline to six months.

In the West, 37 (73%) PTs were at least somewhat familiar with the GMFM-88 at baseline with 7 (14%) indicating they were highly familiar. Nineteen (37%) PTs indicated that they had used the GMFM-88 at least once, with only one PT indicating high use. Following the intervention 46 (98%) PTs in the West were familiar with the GMFM-88, and there was no significant increase in reported use from baseline to six months. Conditional odds ratios for change in familiarity and use of the GMFM-88 are presented in Table 5.

### Familiarity and use of the GMFM-66

The GMFM-66 is an improvement on the GMFM-88 and contains 66 items taken from the original measure. In addition to having fewer items, the GMFM-66 provides interval-level measurement and maintains the strong psychometric properties of the GMFM-88. It has the potential to be a more powerful tool for therapists but it does require a computer to score the items, and it takes time for the user to learn to interpret the item maps.

Sixty-one (86%) therapists in the East reported at least some familiarity with the GMFM-66 with 23 (32%) indicating high familiarity at baseline (Figure 5). Despite this, only 35 (49%) therapists reported using it. Immediately post-intervention, there was a significant increase in the number of therapists indicating they were using the GMFM-66, with 30 (45%) reporting having used it at least once and an additional 24 (36%) reporting high use.

At baseline, 30 (59%) PTs in the West indicated at least some familiarity with the GMFM-66 while 11 (22%) indicated they were using it. Immediately post-intervention, 46 (98%) PTs indicated they were familiar with the GMFM-66, and overall users increased to 31 (66%) with high users increasing from 2 (4%) to 12 (26%). Conditional odds ratios for change in familiarity and use of the GMFM-66 are presented in Table 6.

### Familiarity and use of the motor growth curves

The Motor Growth Curves were originally developed by tracking a large number of children with CP over time using both the GMFCS and the GMFM-66. These curves describe patterns of motor development over time and allow users to predict a child's future motor abilities, given the child's current age and GMFCS level. The motor growth curves are the most recent of the measurement tools, and PTs have had the least amount of time to become comfortable with their use and interpretation.

Although 50 (70%) therapists in the East indicated that they had at least some familiarity with the Motor Growth Curves, only 28 (39%) reported using them (Figure 6). Immediately post-intervention all but one PT in the East indicated they were familiar with the Motor Growth Curves and 51 (76%) indicated they used them.

Sixteen (31%) PTs in the West had some familiarity with the Motor Growth Curves at baseline and only four (8%) indicated using them. Immediately post-intervention, familiarity had increased, with 45 (96%) of the PTs in the West reporting familiarity and 14 (30%) reporting using them at least once with a further 11 (24%) reporting high use. Conditional odds ratios for change in familiarity and use of the Motor Growth Curves are presented in Table 7.

### Long-term impact

The increased familiarity with all the measurement tools reported immediately following the intervention was sustained one year later. With the exception of the GMFM-88, reported use of the tools also increased and the effect remained one year later. Because the GMFM-66 was an improvement to the GMFM-88, therapists who were not familiar with the GMFM-88 would likely go straight to learning and using the GMFM-66. On the other hand, therapists familiar with the GMFM-88 might have continued to use it because there still are indications for use of the GMFM-88 to provide a more detailed assessment of certain children.

### Regional differences

The results indicate that therapists in the East reported greater familiarity and use of the measurement tools at baseline than those in the West, but the intervention still had a significant impact on therapists in both regions. Familiarity with the tools improved significantly following the intervention for both regions. For the GMFCS and the GMFM-66 a time by region interaction was found indicating that the change in familiarity was significantly greater in the West. In terms of using the measurement tools, PTs in the East were more likely than PTs in the West to report high use of the GMFCS (OR = 17.7, 95% CI 4.1 to 76.3) and GMFM-88 (OR = 84.7, 95% CI 11.1 to 644.8), but this was not statistically significant for the GMFM-66 (OR = 1.8; 95% CI 0.6 to 5.3) or the MGCs (OR = 0.4; 95% CI 0.1 to 1.1). PTs in the East were more likely than those in the West to report using the tools at least some of the time: GMFCS (OR = 15.0; 95% CI 1.7 to 134.3), GMFM-88 (OR = 13.1; 95% CI 4.5 to 38.3), GMFM-66 (OR = 3.3; 95% CI 1.5 to 7.4) and the MGCs (OR = 9.9; 95% CI 2.5 to 39.2).

### Organizational characteristics

Table 8 contains details of the descriptive statistics (mean, median) and factor loadings related to organizational characteristics and culture towards research, measurement and evidence-based practice. Factor analysis of the nine items produced a three-factor solution with a combined explained variation of 72%. The final question regarding organizational resistance to change was a noisy item with explained variation of only 18%; it was subsequently removed and the factor analysis repeated.

**Table 8:** Descriptive statistics of organizational characteristics as perceived by individual physical therapists at baseline (n = 122)

			Factor Loadings		
			
**Organizational characteristics: survey items**^**a**^	**Mean (SD)**^**b**^	Median	Resources	Culture	Supervisor
I have access to someone within my organization who can help me to interpret or utilize research evidence	6.1 (2.9)	6	0.62	0.34	nil

My organization supports and/or provides ongoing training in the use of standardized measures	6.1 (2.6)	7	0.76	0.21	0.19

Mechanisms exist in my organization that facilitate the transfer of research evidence into my organization	5.2 (2.6)	5	0.85	0.29	0.21

Overall my organization provides adequate resources (financial or personnel) to implement decisions that are based on research evidence	5.0 (2.4)	5	0.78	0.34	0.26

I frequently hear the terms 'research' or 'research evidence' during policy or program planning discussions within my organization	6.2 (2.4)	7	0.31	0.82	0.23

Overall the culture in my organization is one that highly values the use of research evidence in decision-making for program planning	6.6 (2.5)	7	0.37	0.79	0.29

Research evidence is consistently included in the decision-making process related to program planning in my organization	5.5 (2.3)	6	0.41	0.71	0.41

My direct supervisor expects me to include research evidence in decision-making related to program planning	5.5 (2.6)	6	0.28	0.50	0.83

At my organization, resistance to change is a barrier to using research evidence	3.4 (2.5)	3	removed		

^a ^Items were scored on a 10-point scale from 1 = 'not at all' to 10 = 'to a great extent'

^b ^Standard deviation of the mean

Factor analysis produced a 3-factor solution explaining 72% of the variance. The final question re: organizational resistance to change was a noisy item with an unexplained variance of 82% and was dropped from the model.

The factor analysis yielded the following three main factors: research culture, resources, and supervisor expectation accounting for 78.8% of the total variation. These factors were then included in the models for each outcome and a likelihood ratio test was used to determine if their presence improved the model. The 'resource' factor was not significant for any of the measurement tools and was therefore dropped from the models. Research culture and supervisor expectation were added as fixed effects in the models for the eight outcomes of familiarity and use of the GMFCS, GMFM-88, GMFM-66, and the MGCs for both East and West settings to investigate the impact of these factors on the overall results.

Research culture of the organization had a significant impact on GMFCS familiarity over the six-month intervention (OR = 3.6, 95% CI 1.5 to 8.7) (Table 4). Both research culture (OR = 3.0, 95% CI 1.1 to 7.9) and supervisor expectation for use of measurement tools (OR = 2.6, 95% CI 1.1 to 6.0) were significant predictors in explaining changes in reported use of the GMFCS from 'none' to 'some' and supervisor expectation (OR = 2.0, 95% CI 1.0 to 3.9) when examining the difference between 'some' versus 'high' use. The organizational characteristics did not have a significant impact on the change in familiarity and use of the other tools.

## Discussion

This is the first study we are aware of which evaluates the impact of a multi-faceted KB intervention to help bridge the evidence to practice gap within children's rehabilitation organizations. Following a six-month (two hours/week) KB intervention, changes were reported in practicing PTs' self-reported familiarity with and use of four specific evidence-based measurement tools in two regions of Canada. The changes were maintained one year later. These results are in line with those of a recent systematic review of KT interventions for rehabilitation professionals (OT and PTs) that found moderate level evidence that active multi-component KT interventions (*i.e*., combination of opinion leaders, outreach visits, working groups, printed materials) improved knowledge and practice behaviours compared with passive dissemination strategies for PTs [27].

How our results fit with those of Dobbins *et al*. [31] is less clear. Their randomized control trial involved three KT intervention groups moving from the most passive (access to on-line systematic reviews through healthevidence.ca, or HE), to a moderately interactive KT strategy (access to HE plus receiving tailored, targeted messages, or TM), and the most interactive (HE plus TM plus KBs). There was no significant effect of the intervention for any of the groups on the primary outcome of using evidence in program decisions; however, there was an effect on public health policies and programs for decision makers receiving the moderately interactive approach of HE plus TM. The addition of the KB seemed to wash out the effect of the HE plus TM strategy. Support for the HE plus TM plus KB was found only in those departments where the organizational culture for research was low. Our results showed that a strong research culture and supervisor expectations were the significant factors in predicting familiarity and use of only one of our measurement tools, the GMFCS. The GMFCS is used not only by PTs but by other clinicians as well, and therefore PTs might have had greater expectations to use this tool, especially when communicating with other service providers. Availability of organizational resources were not observed to have a significant impact on familiarity or use of the measurement tools, perhaps because the study team provided educational resources (manuals, CD-ROM training) and financial remuneration for the KBs. The target in the Dobbins study [31] was on individual decision makers in public health departments to increase their capacity to be evidence-based and to impact on health policies and programs. An important detail is that the KB was not situated in the public health departments, and 30% of the health departments never received any of the brokering intervention. In contrast, our study was focused primarily on the implementation of specific evidence-based measurement tools by KBs embedded in the clinical sites on knowledge and on use by practicing clinicians, rather than capacity building which would take longer to show an impact. A large component of our study was the multiple supports offered to KBs throughout the intervention, including facilitation of a KB community of practice, which is thought to be an important component of KT interventions [25,26,31].

The KB model employed in this study was designed to address many of the barriers to knowledge transfer identified in the literature, and was primarily focused on the 'action' phase of moving evidence into practice. An attempt was made to minimize potential barriers by: obtaining support from organizations prior to implementing the KB model; engaging KBs who were enthusiastic about the role; providing financial support to each of the rehabilitation organizations to allow for dedicated time for the KB role; limiting the content to four measurement tools relevant to clinical practice; providing tailored, synthesized materials and training resources in a variety of formats; and providing a social network of support to the KBs comprised of other KBs and the research study team. To implement this KB model in practice without having the evidence synthesized in user-friendly formats would require significantly more time and resources than were provided in this study. This underscores the need for researchers to devote more time to describing and highlighting implications for practice, and to packaging their research materials in ways that make them accessible to front-line practitioners. In addition, academic institutions need to acknowledge the importance of KT activities and to value them in decisions of academic promotion and tenure.

Throughout the study KBs identified the significance of the PT research coordinator or 'broker to the KBs' as being an important facilitator of the process. The research coordinator readily understood the therapists' needs, responded in a timely fashion to KBs requests for help or information and was in contact on a regular basis throughout the intervention with KBs and the study team [36].

Ketelaar *et al*. [25] have demonstrated the knowledge/use gap with a subset of these measurement tools with PTs in the Netherlands. In our study, therapists from the West tended to have a wider gap between familiarity and use of measurement tools at baseline than those in the East, and this KB strategy helped to narrow the gap considerably. Changes reported in rehabilitation organizations across the three provinces imply that the model can be generalized across different practice settings because clinicians provided service to children of all ages, practiced in urban and rural settings, and had varying levels of baseline knowledge. It is encouraging that the changes reported immediately post-intervention were maintained one year later.

A success of this intervention was the high retention rate of 24 out of 25 KBs (96%) and 114 of 122 (93%) participating therapists through the six-month intervention. This is an indication of the interest and enthusiasm that was generated for this KB process.

## Limitations

A before-after research design does not provide a concurrent control group for comparison. However, the tools we evaluated have been published and available to service providers for several years through traditional dissemination methods (journal articles, books, workshops, and the internet), and they are still not routinely being used in clinical practice. We therefore felt that we had a stable baseline from which to make a comparison over time with each site acting as its own control.

The sample size for this study was estimated based on change on the 10-point Likert scale of the survey questionnaire. To facilitate clinical interpretation of the scale and because the data were not normally distributed, the 10-point scale was collapsed into three categories. The lack of precision in some of our odds ratio estimates may have been limited by our sample size.

There is a need for reliable and valid outcome measures to evaluate the impact of KT interventions and to identify and document supports and barriers to implementation. The primary outcome measure in this study was a survey questionnaire used to evaluate PTs' self-reported knowledge of and use of four specific measurement tools. Self-report measures can lead to an over-reporting bias [39]. It would have been useful to validate the reported use in our study through a chart audit. Barwick *et al*. [26] found no difference in reported use of measures between a community of practice group and a control group, but did find a difference in actual use in the community of practice group as measured through chart audit.

Ideally it would also be important to determine whether the observed changes in knowledge and use of the measurement tools impacted child and family outcomes. Through this study KBs and PTs identified an additional barrier that would be important to address prior to looking at the impact on child and family outcomes. They sometimes found it challenging to communicate the results from these measurement tools with families, particularly 'classifying children,' sharing prognostic information with families at a time when families were 'ready' to hear the information, and presenting results in a sensitive manner while supporting families to maintain hope. This information will be useful for developing further resources for therapists and families to help address this important barrier.

## Summary

This multi-centre study showed that by providing modest financial remuneration (two hours/week for six months), ongoing resource materials, and personal and intranet support, a KB embedded within a clinical site was effective in increasing self-reported knowledge and use of specific evidence-based measurement tools. These reported changes were sustained at 12 months. Because the study provided many supports to the organizations, further research is warranted into understanding feasible, cost effective models for implementing a KB strategy to move other measures and evidence based information into clinical practice.

## Competing interests

Three of the authors (DJR, PLR, LMA) receive royalties from the sale of GMFM manuals. DJR and PLR put all proceeds into a research account and none are taken for personal use.

## Authors' contributions

DJR conceived of the study, participated in design, project management, analysis, and drafted the manuscript. LMR provided project management, data analysis, writing, and review of the manuscript. SDW participated in project management, data analysis, and review of the manuscript. PLR, LR, DC, JD, DJB, SEH participated in the research design, project management, and review of the manuscript. LMA participated in data analysis and review of the manuscript. All authors read and approved the final manuscript.

## Authors' Information

DJR is partially supported by research scholar awards from the Ontario Federation for Cerebral Palsy and the McMaster Child Health Research Institute, McMaster University.

## Supplementary Material

Additional file 1 **Table S1: Characteristics of the Motor Growth Measures**. The additional file provides a brief description of the four measurement tools (GMFCS, GMFM-88, GMFM-66 and the Motor Growth Curves) and their measurement characteristics. Click here for file

## References

[B1] Haynes RB What kind of evidence is it that Evidence-Based Medicine advocates want healthcare providers and consumers to pay attention to? BMC Health Serv Res 2002 2 3 10.1186/1472-6963-2-3 11882257 PMC99045

[B2] American Physical Therapy Association Who are physical therapists and what do they do? Phys Ther 2001 81 S31 42

[B3] Palisano RJ Rosenbaum P Walter S Russell D Wood E Galuppi B Development and Validation of a Gross Motor Function Classification System for children with cerebral palsy Dev Med Child Neurol 1997 39 214 223 10.1111/j.1469-8749.1997.tb07414.x 9183258

[B4] Palisano RJ Rosenbaum P Bartlett D Livingstone M Content validity of the expanded and revised Gross Motor Function Classification System Dev Med Child Neurol 2008 50 744 750 10.1111/j.1469-8749.2008.03089.x 18834387

[B5] Russell D Rosenbaum P Cadman D Gowland C Hardy S Jarvis S The Gross Motor Function Measure: A means to evaluate the effects of physical therapy Dev Med Child Neurol 1989 31 341 352 10.1111/j.1469-8749.1989.tb04003.x 2753238

[B6] Russell D Avery L Rosenbaum P Raina P Walter S Palisano R Improved scaling of the Gross Motor Function Measure for children with cerebral palsy: Evidence of reliability and validity Phys Ther 2000 80 873 885 10960935

[B7] Russell D Rosenbaum P Avery L Lane M The Gross Motor Function Measure (GMFM-66 andGMFM-88) User's Manual 2002 London, MacKeith Press

[B8] Avery LM Russell DJ Raina PS Walter SD Rosenbaum PL Rasch analysis of the Gross Motor Function Measure: Validating the assumptions of the Rasch model to create an interval level measure Arch Phys Med Rehab 2003 84 697 705 10.1016/s0003-9993(02)04896-7 12736885

[B9] Rosenbaum PL Walter SD Hanna SE Palisano RJ Russell DJ Raina P Wood E Bartlett DJ Galuppi BE Prognosis for Gross Motor Function in Cerebral Palsy: Creation of motor development curves JAMA 2002 288 1357 1363 10.1001/jama.288.11.1357 12234229

[B10] Russell D Rosenbaum P Lane M Gowland C Goldsmith C Boyce W Plews N Training users in the Gross Motor Function Measure: Methodological and practical issues Phys Ther 1994 74 630 636 8016195 10.1093/ptj/74.7.630

[B11] Lane M Russell D Gross Motor Function Measure (GMFM) Self-instructional Training CDROM [CD-ROM] 2003 London, Mac Keith Press

[B12] Palisano RJ Hanna SE Rosenbaum P Russell DJ Walter SD Wood E Raina P Galuppi B Validation of a model of gross motor function for children with cerebral palsy Phys Ther 2000 80 974 985 11002433

[B13] Russell DJ Leung KM Rosenbaum PL Accessibility and perceived clinical utility of the GMFM-66: Evaluating therapists' judgements of a computer-based scoring program Phys Occup Ther Pediatr 2003 23 3 45 58 12951787

[B14] Russell DJ Gorter JW Assessing functional differences in gross motor skills in children with cerebral palsy who use an ambulatory aid or orthoses: Can the GMFM-88 help? Dev Med Child Neurol 2005 47 462 467 10.1017/S0012162205000897 15991866

[B15] Palisano R Cameron D Rosenbaum PL Walter SD Russell D Stability of the Gross Motor Function Classification System Dev Med Child Neurol 2006 48 424 428 10.1017/S0012162206000934 16700931

[B16] Rosenbaum PL Palisano RJ Bartlett DJ Galuppi BE Russell DJ Development of the Gross Motor Function Classification System for cerebral palsy Dev Med Child Neurol 2008 50 249 253 10.1111/j.1469-8749.2008.02045.x 18318732

[B17] Hanna SE Bartlett DJ Rivard L Russell DJ Reference curves for the Gross Motor Function Measure (GMFM-66): Percentiles for clinical description and tracking over time among children with cerebral palsy Phys Ther 2008 88 596 607 10.2522/ptj.20070314 18339799 PMC2390723

[B18] Gorter JW Ketelaar M Rosenbaum P Helders PJM Palisano R Use of the Gross Motor Function Classification System in infants with cerebral palsy: The need for reclassification at age 2 or older Dev Med Child Neurol 2009 51 46 52 10.1111/j.1469-8749.2008.03117.x 19018834

[B19] Hanna SE Rosenbaum PL Bartlett DJ Palisano RJ Walter SD Avery L Russell DJ Stability and decline in gross motor function among children and youth with cerebral palsy aged 2 to 21 years Dev Med Child Neurol 2009 51 295 302 10.1111/j.1469-8749.2008.03196.x 19391185

[B20] Hanna SE Russell DJ Bartlett DJ Kertoy ML Rosenbaum PL Wynn K Measurement practices in pediatric rehabilitation: A survey of physical therapists, occupational therapists, and speech-language pathologists in Ontario Phys Occup Ther Pediatr 2007 27 25 42 17442653

[B21] Law M King G Russell D MacKinnon E Hurley P Murphy C Measuring outcomes in children's rehabilitation: A decision protocol Arch Phys Med Rehabil 1999 80 629 636 10.1016/S0003-9993(99)90164-8 10378487

[B22] Cochrane LJ Olson CA Murray S Dupuis M Tooman T Hayes S Gaps between knowing and doing: Understanding and assessing the barriers to optimal healthcare J Contin Educ Health Prof 2007 27 94 102 10.1002/chp.106 17576625

[B23] Saleh MN Korner-Bitenskey N Snider L Malouin F Mazer B Kennedy E Roy MA Actual versus best practices for young children with cerebral palsy: A survey of paediatric occupational therapists and physical therapists in Quebec, Canada Dev Neurorehabil 2008 11 60 80 10.1080/17518420701544230 17943507

[B24] Canadian Institutes of Health Research About Knowledge Translation Canadian Institutes of Health Research http://www.cihr-irsc.gc.ca/e/29418.html (Retrieved on July 31, 2010 from

[B25] Ketelaar M Russell DJ Gorter JW The challenge of moving evidence-based measures into clinical practice: Lessons in knowledge translation Phys Occup Ther Pediatr 2008 28 191 206 10.1080/01942630802192610 18846897

[B26] Barwick MA Peters J Boydell K Getting to uptake: Do communities of practice support the implementation of evidence-based practice? J Can Acad Child Adolesc Psychiatry 2009 18 16 29 19270845 PMC2651208

[B27] Menon A Korner-Bitensky N Kastner M McGibbon A Straus S Strategies for rehabilitation professionals to move evidence based knowledge into practice: A systematic review J Rehabil Med 2009 41 1024 1032 10.2340/16501977-0451 19893996

[B28] Canadian Health Services Research Foundation The theory and practice of knowledge brokering in Canada's health system Canadian Health Services Research Foundation http://www.chsrf.ca/brokering/pdf/Theory_and_Practice_e.pdf (Retrieved on January 12, 2010 from

[B29] Dobbins M Robeson P Ciliska D Hanna S Cameron R O'Mara L DeCorby K Mercer S A description of a knowledge broker role implemented as part of a randomized controlled trial evaluating three knowledge translation strategies Implement Sci 2009 4 23 10.1186/1748-5908-4-23 19397820 PMC2680804

[B30] Robeson P Dobbins M DeCorby K Life as a knowledge broker in public health Journal of the Canadian Health Libraries Association 2008 29 79 82

[B31] Dobbins M Hanna SE Ciliska D Manske S Cameron R Mercer SL O'Mara L DeCorby K Robeson P A randomized controlled trial evaluating the impact of knowledge translation and exchange strategies Implement Sci 2009 4 61 10.1186/1748-5908-4-61 19775439 PMC2936828

[B32] van Kammen J Jansen C Bonsel G Kremer J Evers J Wladimoroff J Technology assessment and knowledge brokering: The case of assisted reproduction in The Netherlands Int J Technol Assess Healthcare 2006 22 302 306 10.1017/s026646230605118x 16984057

[B33] Lyons R Warner G Langille L Phillips SJ Piloting knowledge brokers to promote integrated stroke care in Atlantic Canada Evidence in action, acting on evidence: A casebook of health services and policy research knowledge translation stories 2006 Ottawa, Canadian Institutes of Health Research 57 60

[B34] Scott-Findlay S Golden-Biddle K Understanding how organizational culture shapes research use J Nurs Adm 2005 35 359 365 16077278

[B35] Graham ID Logan J Harrison MB Straus SE Tetroe J Caswell W Robinson N Lost in knowledge translation: Time for a map? J Contin Educ Health Prof 2006 26 13 24 10.1002/chp.47 16557505

[B36] Rivard LM Russell DJ Roxborough L Ketelaar M Bartlett D Rosenbaum P Promoting the use of measurement tools in practice A mixed-methods study of the activities and experiences of physical therapist knowledge brokers Phys Ther 2010 90 1580 1590 10.2522/ptj.20090408 20813819

[B37] Fleuren M Wiefferink K Paulussen T Determinants of innovation within healthcare organizations Int J Qual Healthcare 2004 16 2 107 123 10.1093/intqhc/mzh030 15051705

[B38] Hedeker D MIXNO: a computer program for mixed-effects nominal logistic regression J Stat Software 1999 4 5 1 92

[B39] Adams AS Soumerai SB Lomas J Ross-Degnan D Evidence of self-report bias in assessing adherence to guidelines Int J Qual Healthcare 1999 11 187 192 10.1093/intqhc/11.3.187 10435838

[B40] Russell D Rivard L Walter S Roxborough L Cameron D Rosenbaum P Bartlett D Darrah J Hanna S Moving cerebral palsy research into practice: Do knowledge brokers make a difference? Dev Med Child Neurol 2009 51 Suppl. 2 76

[B41] Russell D Rivard L Walter S Rosenbaum P Bartlett D Roxborough L Cameron D Darrah J Hanna S Avery L Knowledge brokers to facilitate evidence-based practice: Long-term follow-up of a successful knowledge translation strategy Dev Med Child Neurol 2009 51 Suppl. 5 2419740207

