# Supplementary material for: Using knowledge brokers to facilitate the uptake of pediatric measurement tools into clinical practice: a before-after intervention study
Source: Implement Sci. 2010 Nov 23;5:92. doi: 10.1186/1748-5908-5-92 (PMC3004810; doi:10.1186/1748-5908-5-92)
Supplement: Additional file 1 — Table S1: Characteristics of the Motor Growth Measures. The additional file provides a brief description of the four measurement tools (GMFCS, GMFM-88, GMFM-66 and the Motor Growth Curves) and their measurement characteristics. [file 1748-5908-5-92-S1.DOC]

Additional File, Table S1

| **Characteristics of the Motor Growth Measures** | | | | |
| --- | --- | --- | --- | --- |
| **Measure** | **GMFCS** | **GMFM-88** | **GMFM-66** | **MGCs** |
| **Description** | 5 level severity classification system | 88-item observational assessment tool | 66-item observational assessment tool | 5 level motor growth curves |
| **Purpose** | Discriminative  Classifies the gross motor function of children and youth with CP | Evaluative  Criterion –referenced clinical measure to evaluate change in gross motor function abilities in children with CP (88 items) | Evaluative  Update to the GMFM-88 using Rasch analysis to reduce the number of items to 66 and provide interval level measurement and plotting of scores on an item map | Predictive  Describe patterns of gross motor development over time in children with CP, by age and GMFCS level |
| **Training/time to learn** | 20 minute training DVD available but not required | Manual and self-instructional CD-ROM  May take 6 hrs+ to learn, practice and establish reliability | Manual, self-instructional CD-ROM, and scoring CD-ROM  May take 6 hrs+ to learn, practice and establish reliability; additional time to learn to score and interpret item maps using the computer program | Not yet determined |
| **Time to Use or Administer** | 3-5 minutes | 45-60 minutes | 30-45 minutes to administer (additional time required to review and interpret) | Not yet determined |
| **Technical requirements** | None | Equipment (mat, bench, toys), access to stairs and testing area | Computer for scoring and equipment as with GMFM-88 | None |
| **Psychometrics** | High inter-rater reliability especially when classifying children 2-12 yrs of age; validity and stability established | Reliability, validity, responsiveness established | Reliability, validity, responsiveness established | Uses two reliable and valid measures to plot GMFM-66 scores by age and GMFCS level |
| **Availability** | Can be downloaded free of charge from www.canchild.ca | Manual and CD-ROM training available for purchase | Manual with computer scoring program and CD-ROM training available for purchase | Can be downloaded free of charge from www.canchild.ca; GMFM Manual required to interpret GMFM-66 scores |
| **First published** | 1997 (Expanded and Revised Version published 2008) | 1989; manual in 1990; manual update 1993. | 2000; manual in 2002 | 2002 |

GMFCS = Gross Motor Function Classification System; GMFM = Gross Motor Function Measure; MGCs = Motor Growth Curves; CP cerebral palsy; DVD = Digital Versatile Disc; CD-ROM = Compact Disc Read-Only Memory
